# Heterogeneity in Lowe Syndrome: Mutations Affecting the Phosphatase Domain of OCRL1 Differ in Impact on Enzymatic Activity and Severity of Cellular Phenotypes

**DOI:** 10.3390/biom13040615

**Published:** 2023-03-29

**Authors:** Jennifer J. Lee, Swetha Ramadesikan, Adrianna F. Black, Charles Christoffer, Andres F. Pacheco Pacheco, Sneha Subramanian, Claudia B. Hanna, Gillian Barth, Cynthia V. Stauffacher, Daisuke Kihara, Ruben Claudio Aguilar

**Affiliations:** 1 Department of Biological Sciences, Purdue University, West Lafayette, IN 47907, USA; lee3100@purdue.edu (J.J.L.); black173@purdue.edu (A.F.B.); apachecop@unal.edu.co (A.F.P.P.); subram64@purdue.edu (S.S.); clauh1@purdue.edu (C.B.H.); barthg@purdue.edu (G.B.); cstauffa@purdue.edu (C.V.S.); dkihara@purdue.edu (D.K.); 2 Purdue Institute for Cancer Research, Purdue University, West Lafayette, IN 47907, USA; 3 Department of Computer Science, Purdue University, West Lafayette, IN 47907, USA; christ35@purdue.edu

**Keywords:** rare genetic disease, Lowe syndrome, OCRL1, phosphatase activity, cellular phenotypes

## Abstract

Lowe Syndrome (LS) is a condition due to mutations in the *OCRL1* gene, characterized by congenital cataracts, intellectual disability, and kidney malfunction. Unfortunately, patients succumb to renal failure after adolescence. This study is centered in investigating the biochemical and phenotypic impact of patient’s OCRL1 variants (OCRL1^VAR^). Specifically, we tested the hypothesis that some OCRL1^VAR^ are stabilized in a non-functional conformation by focusing on missense mutations affecting the phosphatase domain, but not changing residues involved in binding/catalysis. The pathogenic and conformational characteristics of the selected variants were evaluated in silico and our results revealed some OCRL1^VAR^ to be benign, while others are pathogenic. Then we proceeded to monitor the enzymatic activity and function in kidney cells of the different OCRL1^VAR^. Based on their enzymatic activity and presence/absence of phenotypes, the variants segregated into two categories that also correlated with the severity of the condition they induce. Overall, these two groups mapped to opposite sides of the phosphatase domain. In summary, our findings highlight that not every mutation affecting the catalytic domain impairs OCRL1′s enzymatic activity. Importantly, data support the inactive-conformation hypothesis. Finally, our results contribute to establishing the molecular and structural basis for the observed heterogeneity in severity/symptomatology displayed by patients.

## 1. Introduction

Lowe Syndrome (LS) is a rare X-linked genetic disease, also known as Oculo-Cerebro-Renal syndrome of Lowe (OCRL), caused by functional deficiencies of the lipid phosphatase OCRL1 (EC 3.1.3.36) [1,2,3]. Patients show ocular, neurological and renal abnormalities, with kidney failure as the most common cause of reduced life expectancy 30–40 years; National Organization for Rare Diseases (https://rarediseases.org/rare-diseases/lowe-syndrome/) (accessed on 11 March 2023). In addition to LS, certain *OCRL1* mutations are found in Dent-2 (D2) disease patients [4,5,6,7,8], who present a milder condition with prevalence of renal symptoms. 

OCRL1 is one of the enzymes involved in phosphatidylinositol phosphate (PIP) metabolism; therefore, it participates in the regulation of such signaling lipids. PIPs transiently accumulate in discrete areas of different cell membranes, where they act as platforms for the recruitment of specific signaling/trafficking proteins. In particular, OCRL1’s substrate, phosphatidylinositol 4, 5-bisphosphate (PI(4,5)P_2_), is enriched at the plasma membrane where it participates in important cellular events including, but not limited to, Arp2/3-mediated actin nucleation, cell–cell and cell–matrix adhesion, phagocytosis and assembly of endocytic sites [9,10,11,12,13,14,15,16,17,18,19]. Further, PI(4,5)P_2_ is substrate for PI3K for the synthesis of PI(3,4,5)P_3_ and, therefore, for recruitment of the basic components of the Akt signaling pathway [20,21]. Therefore, turnover of PI(4,5)P_2_ by OCRL1 is expected to play an important role in deactivation/regulation of the processes/pathways mentioned above; consequently, inadequate regulation of this lipid in LS is believed to lead to abnormal signaling and protein trafficking, contributing to this disease’s manifestations.

Efforts from our laboratory and others led to the identification of important cellular phenotypes caused by OCRL1 lack of function, including defects in fluid-phase uptake [22,23,24], ciliogenesis [25,26,27,28] and others [29,30,31,32]. However, most research (with exceptions, of course) has been pursued using cell systems without kidney relevance (even when renal failure is the most recurrent cause of patient death) and, for simplicity, on cells/animals lacking OCRL1 (even when most patient mutations lead to expression of altered forms, rather than no protein). 

In addition, and although mutations have been identified all throughout the *OCRL1* gene, exons encoding for the phosphatase domain of OCRL1 constitute a mutational *hot spot* for LS [2,3,7,8,33]. Interestingly, most *OCRL1* patient missense mutations are believed to result in impairment of the activity of the lipid phosphatase OCRL1; however, only about 50% of these changes affect residues involved in binding or modification of the substrate. In fact, some affected amino acids are substantially distant from the catalytic site of the enzyme [33]. In other words, several OCRL1 variants found in patients bear intact binding/catalytic sites, but they still render the protein unable to hydrolyze its substrate PI(4,5)P_2,_ triggering characteristic phenotypes [2,3,4,5,22], including Golgi apparatus fragmentation [22,33]. Besides OCRL1, this indirect functional inactivation has also been observed for other altered proteins [34,35,36] and was found to be due to the presence of mutation-stabilized inactive conformations [35,37]. Therefore, we propose that a substantial number of LS-causative *OCRL1* missense mutations lead to a conformational/protein misfolding disease scenario. Indeed, our results indicate that this is the case for OCRL1 patient variants p.D451G and p.V508D [33].

However, whether specific regions or positions within the phosphatase domain are more sensitive or more tolerant to such changes is unknown. Further, it is not clear what level of deviation from normal protein function and process malfunction is tolerable vs. detrimental for cell and organismal physiology. Neither is it known what cellular phenotypic manifestations are more relevant for predicting impact on the patient’s well-being. Indeed, such missing information could help to explain the complexity of the LS outcomes, and how different mutations cause various degrees of symptom severity in patients. 

Therefore, here, in silico, we predicted pathogenicity and studied the enzymatic activity and phenotypic impact of a subset of OCRL1 patient variants (OCRL1^VAR^) selected to sample different sequence regions of OCRL1′s phosphatase domain. 

Based on enzymatic activity and phenotype induction, the OCRL1 variants were segregated into 2 categories:

Group 1: p.K293E, p.M299I, p.R318H, p.D431N and p.I533T: These variants exhibited normal Golgi apparatus morphology and OCRL1 phosphatase activity. In addition, these OCRL1^VAR^ are classified as benign or involved in milder forms of the disease. 

Group 2: p.I393F, p.D451G, p.V508D and p.Y513C present Golgi apparatus fragmented morphology and lack of OCRL1 phosphatase activity. Interestingly, residues changed in variants from this group cluster, together on one face of the phosphatase domain 3D structure, while group 1 is located on the opposite side. 

In summary, our findings highlight that not every amino acid substitution affecting the catalytic domain impairs OCRL1′s catalytic activity. Importantly, alterations at positions outside the active site can affect the enzymatic activity of OCRL1, supporting the existence of conformational disease components in LS. Overall, our results contribute to determining the molecular and structural basis for the observed heterogeneity in severity and symptoms exhibited by patients.

## 2. Materials & Methods

### 2.1. Reagents and Constructs

Reagents were obtained from Fisher Scientific (Fairlawn, NJ, USA) or Sigma Aldrich (St. Louis, MO, USA) unless stated otherwise. Antibodies used in this study are provided in Supplementary Table S2. Site-directed mutagenesis was carried out with the Quikchange Lightning mutagenesis kit (Agilent Technologies, Santa Clara, CA, USA), and pEGFP-c1 *hsOCRL1* (wild-type, isoform b), and pNIC-CH2-*hsOCRL1* (wild-type, amino acids 215–563) were used as templates to create various, mutant *OCRL1* constructs reproducing missense variants, as listed in Supplementary Table S3. 

### 2.2. Cells and Culture Conditions and Transfections

Normal human proximal tubule epithelial (HK2) and human embryonic kidney epithelial 293T (HEK293T) and 293A (HEK293A) cells were purchased from ATCC. *OCRL1* Knock-out (KO) HK2 and HEK293T cells were described earlier [25]; KO cell lines were previously validated [25,30]. All cells were cultured in DMEM with streptomycin/penicillin, 2 mM L-glutamine and 10% fetal bovine serum (FBS) and maintained in an incubator at 37 °C with 5% CO_2_. For the expression of OCRL1, wild-type and mutant constructs, cells were transfected with FuGENE reagent (FuGENE 6 at a 6:1 reagent:DNA ratio or FuGENE 4K at a 4:1 reagent:DNA ratio, based on reagent availability (Promega)), following manufacturer protocols. In short, FuGENE was added to 100 μL of serum-free DMEM media, briefly vortexed, and incubated at room temperature for 5 min. Then, 1 μg of DNA was added to the reaction tube, briefly vortexed, and incubated at room temperature for 30 min. The final complex solution was added dropwise to the cells, and the plate was swirled to evenly spread the solution within the cell-containing wells. 

### 2.3. Western Blotting

HK2 *OCRL1* KO cells were seeded onto 6-well plates and grown to 60–70% confluency in complete media. After a 24-h transfection period with 1 μg DNA for OCRL1 expression, the cells were washed with cold PBS and collected by mechanical scraping with 50 µL/well of lysis buffer (50 mM Tris at pH 7.4, 150 mM NaCl, 0.01% Triton X-100, 0.1% sodium dodecyl sulfate, 0.5% sodium deoxycholate, and 2X EDTA-free protease inhibitors). Lysates were maintained on ice for 30 min and centrifuged at 12,000 rpm for 15 min at 4 °C. Supernatant fractions were collected and analyzed by SDS-PAGE using 10% poly-acrylamide gels, which were later transferred to nitrocellulose membranes. Membranes were blocked using 5% skim milk in PBST and immunoblotted with primary antibodies overnight at 4 °C and HRP-conjugated secondary antibodies for 45 min at 25 °C.

### 2.4. Immunoprecipitation of Full Length OCRL1^WT/VAR^

HEK293A cells were seeded on 150 mm tissue culture dishes and transfected with 12 μg DNA for OCRL1 constructs for 24 h. Cells were then washed with cold PBS and collected by mechanical scraping with 500 µL/plate of lysis buffer (200 mM Tris at pH 7.4, 100 mM NaCl, 5% glycerol, 2X EDTA-free protease inhibitors). Lysates were kept on ice for 20 min and centrifuged at 13,000 rpm for 30 min at 4 °C [38,39]. Supernatant was collected and added to GFP-Trap Agarose (ChromoTek, Planegg, Germany) for immunoprecipitation following the manufacturer’s protocol. Briefly, supernatant was incubated at 4 °C for 1 h in an end-over-end rotator. Beads were then washed twice with base buffer (200 mM Tris at pH 7.4, 100 mM NaCl, 5% glycerol) and twice with assay buffer (299 mM Tris at pH 7.4, 100 mM NaCl, 2% glycerol, 20 mM MgCl_2_), with centrifugation at 2500× *g* for 5 min at 4 °C. 

### 2.5. Purification of Bacterially Produced OCRL1^WT/VAR^ Phosphatase Domain

The phosphatase domain of wild-type human OCRL1 (215–563 amino acids) was cloned in a pNIC plasmid that contains an N-terminal Histidine tag containing 6 histidine residues in tandem [40]. Using site-directed mutagenesis, missense mutations were introduced for respective mutants under study. Plasmids were transformed in Rosetta (DE3) competent cells. Bacterial cultures were grown overnight at 37 °C, in LB medium supplemented with 2.5% glucose, 1X kanamycin, and 1X chloramphenicol. The following day, cultures were expanded in super broth media containing 1X kanamycin, and 1X chloramphenicol for 3 h at 37 °C. Then, cultures were supplemented with 0.1 mM IPTG and incubated for 5 h at 30 °C.

Cells were harvested by centrifugation (3000× *g*, 10 min), and pellets were stored at −80 °C until use. Cells were resuspended and lysed in lysis buffer containing 200 mM Tris pH 7.4, 10% glycerol, 500 mM NaCl, 0.1% Tween 20, 1 mg/mL lysozyme and EDTA-free protease inhibitor. Cells were disrupted by sonication at 60% power for three sets of 33 pulses with 30 s breaks in between pulses. Cell debris was removed via centrifugation at 21,500× *g*, 30 min, 4 °C [33]. The supernatant was transferred to tubes containing His bind resin (Millipore Sigma, Burlington, MA, USA, Catalog number 69670) and incubated at 4 °C on a shaker (12 rpm) for 2 h. Beads were washed four times with two washes each of 20 mM imidazole and 40 mM imidazole, both of which were prepared in the lysis buffer excluding lysozyme and protease inhibitor and pH 7.4. Then, the protein was eluted with 1 M Imidazole pH 7.4 by incubating for 2 h at 4 °C on a shaker (12 rpm). Supernatant (after centrifugation at 1000× *g*, 2 min) was loaded onto desalting columns (Thermo Scientific, Waltham, MA, USA, Zeba, 89891) and centrifuged (1000× *g*, 2 min, acceleration: 5), and purified protein was obtained. The desalting columns were pre-washed three times with desalting buffer containing 200 mM Tris, pH 7.4, 2% glycerol, and 300 mM NaCl before each use. Protein concentration was estimated using absorbance at 280 nm on the NanoDrop 1000 (Thermo Fisher, Waltham, MA, USA), as well as by Western blotting and quantitative densitometry [30].Protein concentrations were normalized to that with the lowest yield. Protein was either used immediately for malachite green phosphatase assays, or flash frozen with liquid nitrogen and stored at −80 °C until use.

### 2.6. Malachite Green Phosphatase Assays

For 5′ phosphatase activity assays, the malachite green phosphate assay kit (MAK307, Sigma-Aldrich, St. Louis, MO, USA) was used [33,40,41]. Briefly, for bacterially purified protein, in a 384-well plate, 10 μL of purified OCRL1 (wild type and variant) was incubated for 15 min at room temperature with 1 μL of 10 mM MgCl_2_ and 10 μL of 60 μM PI(4,5)P_2_ diC8 (P-4508, Echelon Biosciences, Salt Lake City, UT, USA) prepared in the same buffer as the purified phosphatases, containing 200 mM Tris, pH 7.4, 2% glycerol, and 300 mM NaCl. To stop enzyme reaction, 20 μL of 0.25X malachite green reagent was added to the reaction wells. After 10 min of color development, absorbance was measured at 620 nm. A standard phosphate curve was prepared (as per manufacturer’s instructions) in the enzyme buffer solution to determine the amount of free phosphate released by the enzyme variants tested. Experiments were repeated at least thrice, and each condition was tested in triplicate. Student’s t-test was used to determine statistical significance.

For phosphatase assays using full-length, mammalian purified protein, GFP-trap immunoprecipitated protein was resuspended in the final volume of 150 µL of assay buffer. In microfuge tubes containing 40 µL of 60 µM PI(4,5)P_2_ diC8 substrate, 40 µL of resuspended protein was added and incubated for 5, 15, or 30 min at room temperature with frequent and gentle agitation [38,39]. Beads were subsequently centrifuged at 2500× *g* for 5 min at 4 °C, and 20 µL of the supernatant was added into three wells (in a 384-well format) containing 20 µL of 0.25× malachite green reagent. After color development for 10 min, absorbance was measured at 620 nm. A standard phosphate curve was prepared in the assay buffer solution. 

### 2.7. Indirect Immunofluorescence and Fluorescence Microscopy

Immunofluorescence procedures were carried out with antibodies diluted in DMEM media supplemented with 10% FBS (as a blocking agent) and 0.1% saponin (as a permeabilizing agent) [25,30,33]. Briefly, upon a 15 min fixation with 4% formaldehyde, cells were incubated with primary antibodies for 1 h at room temperature. Secondary antibodies with fluorescent probe conjugation were incubated for 45 min at room temperature in the dark. DAPI was used to label the nucleus, and coverslips were mounted on glass slides using Fluoromount-G (0100-01, SouthernBiotech, Birmingham, AL, USA). 

All fluorescence imaging was carried out with a Zeiss Axiovert 200 M microscope along with a Zeiss Axiocam MRm monochrome digital camera and the Carl Zeiss Axiovision image acquisition software (version 4.4) [25,30,33]. Random fields of the entire coverslip were imaged, and exposure times for each channel were kept consistent within independent experiments. Total magnification for images were kept at 400× using the Zeiss Objective EC “Plan-Neofluar” 40×/0.75 M27.

### 2.8. Golgi Apparatus Fragmentation Phenotype

Cells were seeded onto glass coverslips in 6-well plates at 60% confluency and transfected with 1.5 μg of respective plasmids for expression of WT and mutated OCRL1. After 24 h, cells were fixed and immunostained with an antibody against a Golgi apparatus marker (TGN46). Transfected cells were imaged and analyzed using the ImageJ software. The TGN46 area of a given transfected cell was outlined and measured using the freehand selection tool [30,33]. Next, the total cell area was outlined and measured using the free-hand selection tool in the GFP channel. The values obtained were divided to obtain the Golgi apparatus area as a fraction of the total cell area for a minimum of 150–200 cells to determine the fragmentation phenotype. These values were compared with the wildtype OCRL1 control. 

### 2.9. In Silico Analysis of OCRL1 Variants

In silico *pathogenicity prediction*. Human genome reference GRCh38 (and corresponding GRCh37) coordinates for all the missense variants used in this study were obtained from the UCSC genome browser (https://genome.ucsc.edu/index.html) (accessed on 11 March 2023) [42]. Where appropriate and required, *OCRL1* canonical transcript (RefSeq ID NM_000276.4 or ENSEMBL ID ENST00000371113.4) was used as input for pathogenicity prediction. For predictions requiring the translated protein sequence, the protein encoded by the canonical transcript (UNIPROT ID Q01968) was used. 

For estimating intolerance to residue substitution in the context of the protein landscape, MetaDome web server (https://stuart.radboudumc.nl/metadome/) (accessed on 11 March 2023) [43] was used. The above specified canonical transcript was used for subsequent analysis. Combined Annotation Dependent Depletion (CADD) score was determined from the CADD web server (https://cadd.gs.washington.edu/) (accessed on 11 March 2023) [44] using GRCh 38 coordinates of the different missense variants. MutPred2 scores were computed on the MutPred2 web server (http://mutpred.mutdb.org/#qform) (accessed on 11 March 2023) [45] using the translated OCRL1 protein sequence obtained from UniProt. PolyPhen2 and SIFT scores were obtained from respective web servers (PolyPhen2: http://genetics.bwh.harvard.edu/pph2/ (accessed on 11 March 2023) [46], SIFT: https://sift.bii.a-star.edu.sg/www/SIFT_seq_submit2.html (accessed on 11 March 2023) [47]) by using the protein sequence (UniProt ID Q01968) in FASTA format. For SIFT, search databases included UniProt-SwissProt + TrEMBL 2010_09 to maximize the number of similar protein sequences used for prediction. To generate MutationTaster predictions, variants of interest were input in the MutationTaster web server (https://www.mutationtaster.org/) (accessed on 11 March 2023) [48,49], followed by selection of the *OCRL1* canonical transcript (see transcript ID above). Variant position information was provided with respect to the coding sequence (open reading frame). REVEL scores for selected *OCRL1* variants were downloaded from dbnSFP database (http://database.liulab.science/dbNSFPconn) (accessed on 11 March 2023) [45,50]. For predicting the effects of missense mutations on OCRL1 phosphatase domain stability, Site Directed Mutator, a web-based tool, was used (http://marid.bioc.cam.ac.uk/sdm2/prediction) (accessed on 11 March 2023) [51]. Briefly, the solved crystal structure of OCRL1 phosphatase domain (PDB ID: 4CMN) served as the wild-type protein structure. Subsequently, missense variants were then mapped on to protein chain A (as provided in RCS PDB structure) to determine the stability of the altered protein.

*Modeling and Molecular Dynamics*. Models were generated using AlphaFold [52] for both the OCRL1 wild type (WT) and all the studied protein variants, with emphasis on the region encoded by amino acids R219 to N559 that was considered the phosphatase domain in isolation. For the WT and variants, an independent single-replicate molecular dynamics (MD) simulation was performed utilizing NAMD 3.0 [53] using the CHARMM36 force field for proteins [54] and a Generalized Born implicit solvent. After equilibration, each simulation was run for 33 ns with a 1 fs time step, at a temperature of 310 K. Analysis was performed using MDAnalysis 2.2.0 [55], sampling the trajectory at intervals of 10 ps.

### 2.10. Statistical Analysis

The analysis was performed as described below and in Taylor, 1997 [56]. When appropriate, the magnitude of errors associated with values derived from algebraic operations using experimentally measured quantities were calculated following standard rules of error propagation (e.g., phosphatase activity results). Statistical significance of differences between RMSF distributions were analyzed using the Kolmogorov-Smirnov test. The student’s t-test was used to evaluate differences among normally distributed phosphatase activity data from recombinant phosphatase domains, while the Wilcoxon’s test was used to evaluate the significance between non-normal Golgi apparatus fragmentation values. Bonferroni’s correction for multiple comparisons was performed whenever applicable (α_C_ = p/n; n being the number of comparisons).

After carefully analyzing each data set distribution the most appropriate representation of data in each case was adopted. These representations included scatter-line and box plots, as they allow thorough examination of the data distribution [56]. All box-plots used in this study represent data distributions with box limits (Inter-quartile range, IQR) at the 25th (Q1) and 75th (Q3) percentiles with the median (Q2) indicated inside the box. The lower and upper whiskers represent the minimum (Q1−1.5×IQR) and maximum (Q3 + 1.5×IQR), respectively. When the data presented a normal distribution, bars representing average with their corresponding standard deviations were used.

## 3. Results and Discussion

### 3.1. OCRL1 Mutant Selection and In Silico Analysis

**Figure 1:**
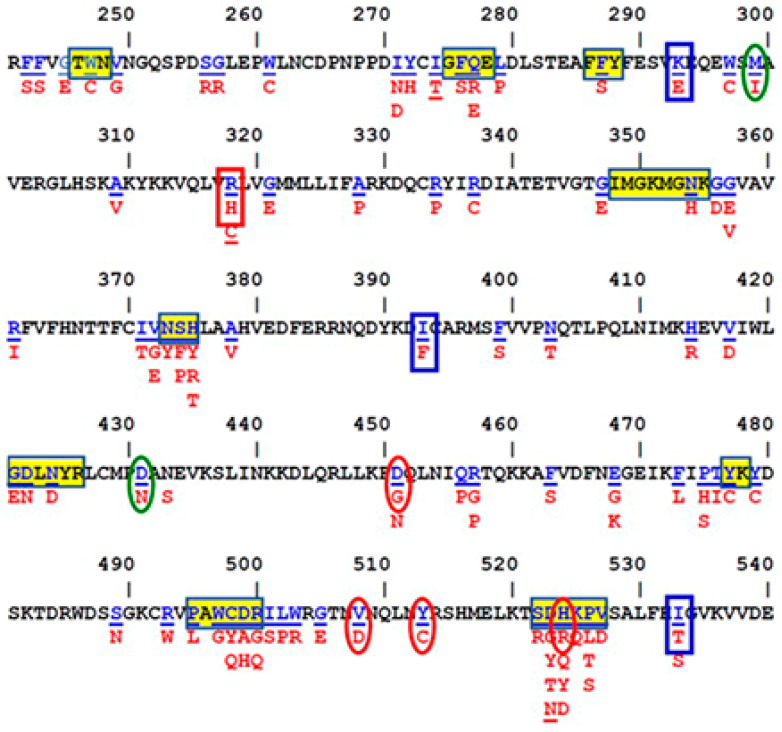
**Missense changes affecting OCRL1′s phosphatase domain used in this study**. The amino acid sequence of the OCRL1 phosphatase domain is shown with underlined blue residues to indicate those affected by missense mutations; amino acids resulting in such mutations are shown in red. Yellow rectangles enclose regions involved in processing/recognition of the substrate. Substitutions exhibited by variants used in this study are shown; those causing LS, D2, and LS/D2 are shown within red ovals, blue and red vertical rectangles, respectively; mutations with uncertain disease significance are shown within green ovals.

We selected *OCRL1* missense mutations affecting different regions of the phosphatase domain, but without altering residues within the catalytic site (Figure 1). If some of these variants would exhibit a decrease in enzymatic activity, it would support the hypothesis that some residue changes can induce detrimental conformational effects on the active site. In other words, some Lowe syndrome patients exhibit a conformational disease scenario.

Pathogenesis analysis: we selected several reported OCRL1 variants (OCRL1^VAR^) bearing different residue substitutions affecting various regions of OCRL1′s phosphatase domain (but without altering the catalytic site, see Figure 1). Table 1 lists the selected variants along with a summary of the in silico predictions of the impact of the reported mutations on OCRL1′s pathogenicity (see Supplementary Table S1 for a complete list of results).

Briefly, we used the following tools (with algorithms based on independent prediction strategies) to perform the analysis: CADD [44], REVEL [45], MutPred2 [57], MutationTaster 2021 [48,49], PolyPhen-2 [46] and SIFT [47]. These tools were selected considering reliability and wide acceptance in the field; for example, several have been included in the guidelines for interpretation of sequence variants by the American College of Medical Genetics (ACMG) and Genomics and the Association for Molecular Pathology [58].

A high confidence conclusion emerged concerning the OCRL1^M299I^ and OCRL1^D431N^ variants as being benign (Table 1). Indeed, the reported relatively high frequency of these OCRL1^VAR^ suggest that they may represent variations in the normal population (see Supplementary Table S1). Therefore, it is not surprising that these variants are classified as likely benign/benign in ClinVar (ACMG classification criteria BP4).

All other analyzed variants were predicted to be pathogenic (Table 1) or deleterious/probably damaging (Supplemental Table S1). However, some are associated with D2 disease, while others with LS, or both. Although different predictions carried different scores and confidence, it was difficult to predict whether individual variants were likely to lead to severe or mild scenarios (Supplementary Table S1). Indeed, some tools gave similar scores to different OCRL1^VAR^ even when some of them were causing D2 and others LS (Supplemental Table S1). In addition, these tools do not provide insights into the molecular mechanism by which all these variants would be pathogenic.

**Figure 2:**
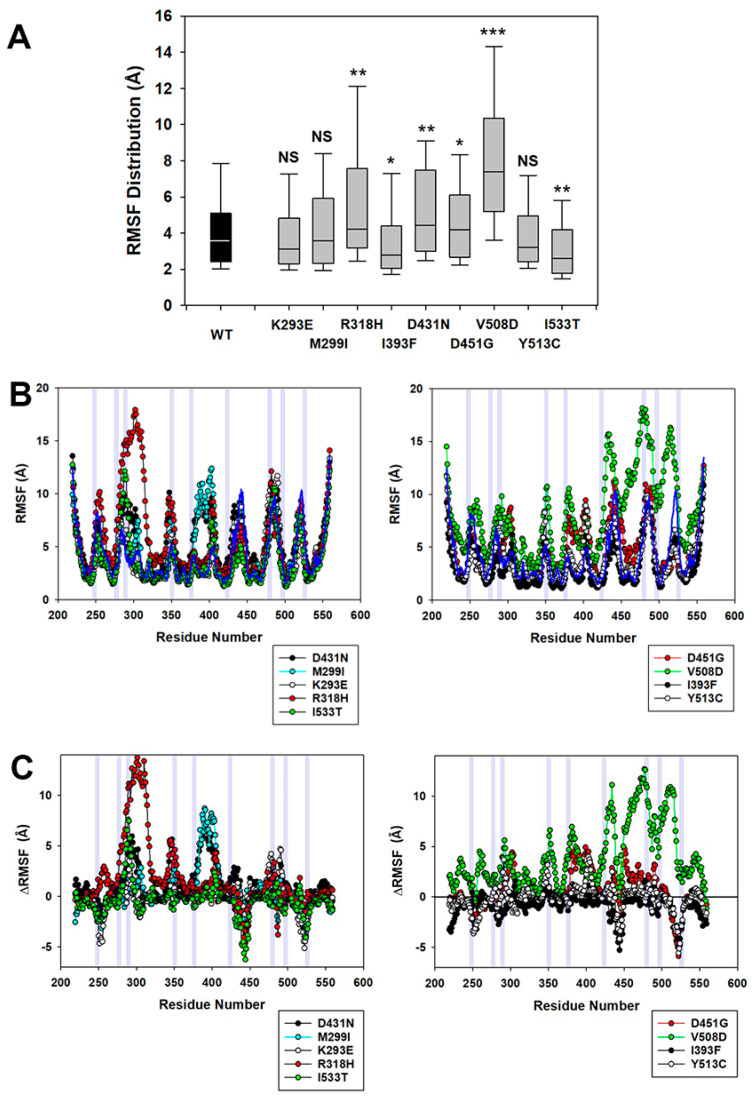
**Molecular Dynamics Analysis of OCRL1 variants.** (**A**) Box plots represent the distribution of individual residue fluctuations measured as RMSF throughout the phosphatase domain of OCRL1^WT^ and the indicated OCRL1^VAR^. All distributions were statistically compared to that corresponding to OCRL1^WT^, using the Kolmogorov–Smirnov test with *p* < 0.05; applying Bonferroni’s correction for nine comparisons; α_B_ ≤ 0.005 (*), α_B_ ≤ 0.001 (**) and α_B_ ≤ 0.0001 (***) for individual comparison. NS: Not significant. (**B**,**C**) Panels show RMSF (**B**) and ∆RMSF (**C**) values for the indicated OCRL1^VAR^ at individual residue positions. For clarity reasons, values for OCRL1^VAR^ were plotted in two graphs shown (left and right) with ≤5 variants per graph bearing substitutions as indicated by the symbol legend. WT distribution (**B**) is indicated by a dark blue continuous line. Grey shaded vertical areas highlight regions within the phosphatase domain involved in substrate binding or catalysis. See text for more details.

Molecular Dynamics (MD) Analysis: we modeled the effects of the residue substitutions K293E, M299I, R318H, I393F, D431N, D451G, V508D, Y513C, and I533T on the structural dynamics of the phosphatase domain of OCRL1 using the known crystal structure of the WT variant as starting point (PDB file 4CMN; [40]). We used the root-mean-square fluctuation (RMSF) as an indicator of individual residue mobility (fluctuation) during simulation. This quantity was calculated for each residue within the domain as described in Materials and Methods, and is shown for each variant in Figure 2. 

We observed that the WT phosphatase domain had an overall RMSF median of 3.31 Å (Figure 2A) within a range of (1.50; 13.49 Å) values for individual residues (Figure 2A,B). As compared to WT, variants such as p.V508D and p.R318H had higher overall fluctuation RMSF medians of 7.35 Å and 4.22 Å within ranges of (2.52; 18.16 Å) and (1.86; 17.93 Å), respectively. In contrast, other OCRL1^VAR^ showed less fluctuation than OCRL1^WT^, for example p.I393F (median: 2.57 Å) and p.I533T (median: 2.75 Å) with identical (1.11; 10.8 Å) ranges (Figure 2A). To convey these general observations, we compiled the ratio between the RMSF medians of each OCRL1^VAR^ and that from WT (Normalized median fluctuation—see Table 1).

To facilitate the visualization of changes in specific residue mobility, we calculated the difference between the RMSF of every variant and WT (∆RMSF) at each residue position (Figure 2C). In all cases, regions involved in substrate binding/catalysis (or close neighboring sequences) were affected by fluctuation changes (grey shaded areas in Figure 2B,C). Therefore, the feasibility of mutation-driven conformational impact on the enzymatic function of the variant is supported by MD data. However, and surprisingly, even the benign p.M299I and p.D431N variants displayed some mobility changes in such functional regions. This observation strongly suggests that some of these alterations can be tolerated at a cellular/organismal level. Further, these benign variants also differed from the WT protein, as they showed a substantial increase in fluctuation, mainly associated with the segment of sequence located between residue positions 375 and 410, which are not directly involved in substrate binding/catalysis (Figure 2C).

Interestingly, some variants such as p.V508D exhibited large residue fluctuations in widespread regions of the active site, despite being located on the spatially opposite side of the phosphatase domain (Figure 2C). This is again consistent with our hypothesis that some patient variants undergo a conformational change that affects function.

We also relied on the use of the Missense3D algorithm [59] to evaluate the impact of the mutations on the domain structure in comparison to WT (PDB 4CMN [40]). The corresponding results indicated no major structural damage for the selected variants (Table 1), except for the phosphatase domain bearing the D451G permutation. In that case, the algorithm predicted some structural damage due to the breakage of a buried H-bond and a salt bridge, along with a replaced buried charge. As expected, all variants exhibited a certain degree of disturbance in positions neighboring the mutated residue and in the regions that were predicted to change mobility by our MD studies (see above).

In addition, variants p.D451G, p.V508D (both known to lack phosphatase activity when the isolated domains expressed in bacteria were tested [33]) and p.Y513C displayed change in the spatial orientation of critical residues involved in enzymatic activity, such as residue R500 [60,61] or others immediately adjacent.

Indeed, our own independent evaluation of the impact of mutations on the structure of the phosphatase domain of WT OCRL1 (PDB file 4CMN) led us to similar conclusions as the predictions described above (Supplementary Figures S1–S5). In addition, we also noted that the loss of a local hydrogen-bond network caused by the D451G change would produce a discontinuity in the structure directly affecting R500, which in turn is in direct connection with the catalytically crucial H524 residue (Supplementary Figure S2). Therefore, we concluded that the D451G change would effectively induce a misalignment of the active site. Similarly, the OH group of Y513 binds E468 and L501, which are embedded in or flanking the catalytic region. Therefore, we expect that p.Y513C variant will also exhibit a conformation-driven impairment of the enzymatic function (Supplementary Figure S4). Finally, the V508 residue is tightly packed against residues flanking the catalytic region (e.g., W503); introduction of a charged D at this position would surely affect packing, being highly disruptive for the domain in general and the active site in particular (Supplementary Figure S3). 

As a whole, our results strongly suggest that some variants (e.g., p.D451G, p.Y513C and p.V508D) display conformational disturbances that affect the catalytic site, while others (e.g., p.M299I, p.D431N and p.I533T) do not seem to carry highly disruptive changes.

To test the validity of in silico predictions and own analyses, we next proceeded to experimentally measure the biochemical and cellular functions of the selected OCRL1^VAR^.

### 3.2. OCRL1^VAR^ Phosphatase Activity

**Figure 3:**
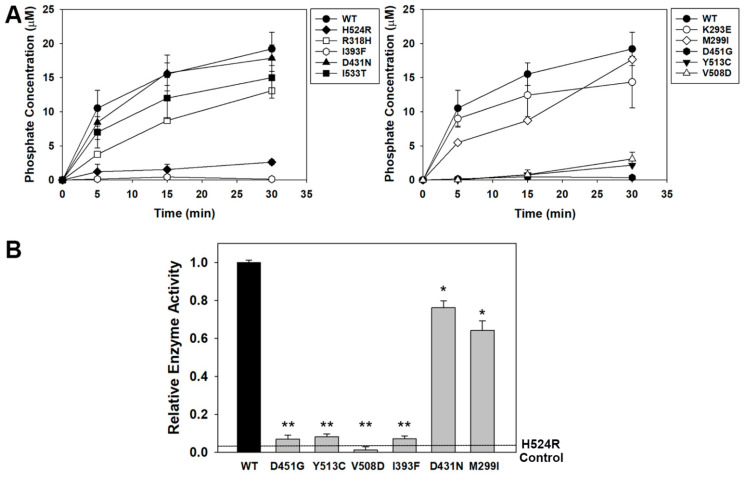
**Phosphatase activity displayed by OCRL1^WT/VAR^.** Phosphatase activity of the selected variants was measured utilizing the malachite green assay, as detailed in Materials and Methods, and using Full length OCRL1^WT/VAR^ immunoprecipitated from human cells (**A**) or the variant’s recombinant, isolated phosphatase domain produced in and purified from bacteria (**B**). (**A**) The biologically relevant, full length indicated OCRL1 variants were tested for their ability to catalyze the release of inorganic phosphate from PI(4,5)P_2_ as a function of time. The phosphatase dead variant pH524R was used as negative control. (**B**) Experiments using an isolated domain were performed to confirm the detrimental direct effect of the missense mutations on the enzymatic activity of the variant’s catalytic domain. Statistical significance of the differences between normalized catalytic activity of OCRL1^VAR^ and WT was assessed using the *t*-test with *p* < 0.05, applying Bonferroni’s correction for six comparisons: α_B_ ≤ 0.008 (*) and α_B_ ≤ 0.001 (**) for individual comparison.

We measured the release of inorganic phosphate as a consequence of OCRL1′s phosphatase activity on its substrate PI(4,5,)P_2_ using the malachite green (MG) colorimetric method [14,33,38,39,40,41,62]. We relied on the use of (1) Full Length (FL) OCRL1^WT/VAR^ purified from a human cell line and (2) isolated recombinant phosphatase domain of OCRL1^WT/VAR^ produced in bacteria and purified (see Materials and Methods) (Figure 3A,B, respectively) to conduct the following assays.

To evaluate the phosphatase activity of GFP-FL OCRL1^VAR^, we immunoprecipitated the different variants expressed in 293A cells using GFP-Trap (Proteintech ChromoTek, see Materials and Methods—see also Supplementary Figure S6 for examples of OCRL1^WT/VAR^ expression/stability *in cells* and captured by GFP-trap, as evaluated by Western blotting). Following washes, beads retaining the GFP-FL OCRL1 variants were tested for phosphatase activity by incubating with 30 µM PI(4,5)P_2_ (diC8: short carbon chain, Echelon Inc., Salt Lake City, UT, USA) for the indicated amount of time (Figure 3A) and phosphate release was measured using the MG assay (see Materials and Methods for details). Results indicate that OCRL1^VAR^ variants segregated into two groups: one displaying phosphatase activity towards the substrate and another one lacking such enzymatic capability (Figure 3A). As expected, the phosphatase-dead OCRL1^H524R^ patient variant (negative control) only produced background levels of signal, while OCRL1^WT^ (positive control) had a measurable phosphate production rate. OCRL1^VAR^, which we had predicted to be benign or represent population variations (p.M299I and p.D431N, Table 1), showed similar phosphatase activity to OCRL1^WT^ (Figure 3A). OCRL1^K293E^, OCRL1^I533T^ and OCRL1^R318H^ associated with D2 and in one case with LS [5,6,63,64] (Table 1), also exhibited substantial phosphatase activity against PI(4,5)P_2_, although perhaps at slightly lower levels (Figure 3A) compared to the phosphatase-dead p.H524R control. Variants p.D451G, p.V508D, p.I393F and p.Y513C showed very low or undetectable catalytic activity (Figure 3A).

The enzymatic activity of some isolated phosphatase domains from activity-impaired OCRL1^VAR^ was also monitored as described before [33]. Since we observed similar results (Figure 3B), these findings strongly suggest that the observations obtained using OCRL1^VAR^ expressed in human cells (see above) are due to a direct effect of the residue change on the function of the catalytic domain of the FL variants. 

### 3.3. Golgi Apparatus Fragmentation Phenotype

**Figure 4:**
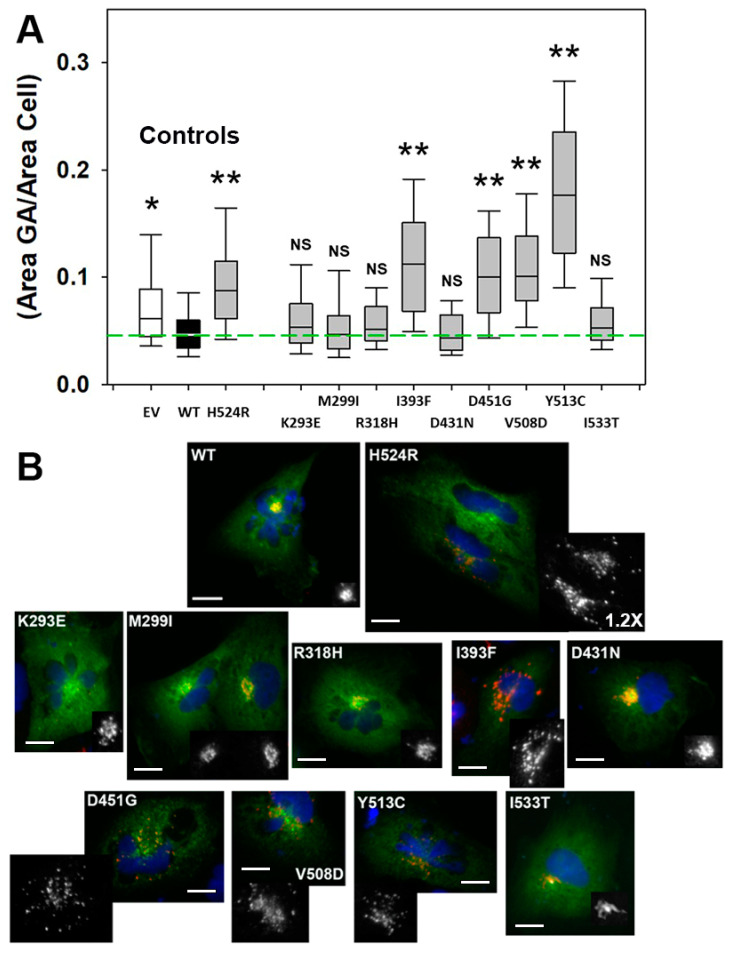
**Golgi apparatus fragmentation phenotype induced by OCRL1^VAR^.** (**A**) Presence and magnitude of this phenotype was assessed by computing the ratio between the area occupied by the Golgi apparatus (GA) and that corresponding to the whole cell (see text for details). Plot depicts combined data from 3 experiments (see Figure 5 for a representative experiment showing individual cell variation). Green dashed line highlights median value obtained for cells expressing OCRL1^WT^ from a distribution with Q1 = 0.034, Q3 = 0.06; IQR = 0.026 (see Materials and Methods). EV: cells transfected with pEGFP empty vector. Statistical significance of the differences between the distributions of WT and variants was assessed using the Wilcoxon test with *p* < 0.05 applying Bonferroni’s correction for multiple comparisons to α_B_ ≤ 0.005 (*) or α_B_ ≤ 0.001 (**) per comparison NS: Not significant. (**B**) Representative images showing HK2 *OCRL1* KO cells expressing GFP-OCRL1^WT^ or the indicated GFP-OCRL1^VAR^ (green signal) and immunostained for the GA marker TGN46 (red signal). The latter is also reproduced in black and white for clearer visualization. Scale bars: 15 µm. When applicable, additional magnification is indicated (i.e., 1.2×).

**Figure 5:**
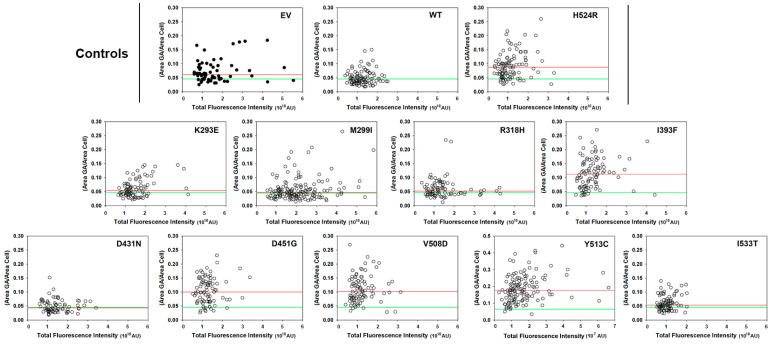
**Golgi apparatus fragmentation phenotype induced by OCRL1^VAR^ as function of each variant intracellular content.** Results in a representative experiment for the evaluation of GA fragmentation induced by the indicated OCRL1^VAR^. The ratio (Area GA/Area cell) was estimated as described in Material and Methods and Figure 4. 150–200 cells HK2 *OCRL1* KO cells expressing only GFP (i.e., transfected with pEGFP empty vector, EV) or GFP-OCRL1^WT/VAR^ were analyzed per sample while also measuring their total fluorescence intensity in arbitrary units reporting total amount of the indicated protein present in the corresponding cells). Green horizontal dashed line depicts the median value obtained for cells only expressing OCRL1^WT^, while the red reference line corresponds with the median value measured for each specific OCRL1^VAR^.

We previously found that Golgi apparatus morphology defects (consisting of a dispersed vesiculated distribution of the organelle in contrast to the compact perinuclear pattern observed with OCRL1^WT^) are associated with OCRL1′s 5′-phosphatase activity deficiency [33]. Therefore, the presence/absence of this phenotype in OCRL1^VAR^-expressors constitutes an obvious *in cell* readout to complement our in vitro enzymatic activity experiments (Figure 3). Briefly, HK2 *OCRL1* KO cells expressing GFP-OCRL1^WT^ or GFP-OCRL1^VAR^ were fixed and immunostained for the trans-Golgi network (TGN) marker TGN46, washed, mounted and imaged (see details in Materials and Methods). At least 150–200 cells were analyzed per sample in each experiment and this was repeated at least thrice. Results were processed as described in Materials and Methods and in Ramadesikan et al., 2021 [33] and presented as the ratio area occupied by the Golgi complex/whole area of the cell (Figure 4 and Figure 5). These values were also plotted vs total GFP fluorescence intensity of individual cells (as an indicator of total amount of OCRL1^WT/VAR^ present) (Figure 5). It should be noted, nevertheless, that Golgi apparatus fragmentation did not vary substantially with the total amount of protein expressed (Figure 5).

Our results show that, as expected, variants p.K293E, p.M299I, p.R318H, p.D431N and p.I533T, with measurable phosphatase activity displayed normal Golgi complex morphology. In contrast, OCRL1^VAR^, with deficient catalytic activity (p.I393F, p.D451G, p.V508D and p.Y513C), exhibited fragmentation of the Golgi apparatus (Figure 4 and Figure 5). Figure 4B provides representative images of OCRL1^VAR^-expressing cells used for this phenotypic analysis.

Interestingly, we also tested known alternative missense mutations affecting the same position yielding different substitutions, (e.g., D451G and D451N; R318H and R318W, R318C, R318L, R318S; I533T and I533S, etc.) and found no difference at all for the induction (or lack thereof) and severity of this phenotype (Supplemental Figure S7).

Strikingly, we observed that, although *OCRL1* KO cells showed Golgi complex fragmentation, some OCRL1^VAR^ (i.e., p.H524R, p.I393F, p.D451G, p.V508D and p.Y513C) exhibited more severe phenotype than cells lacking OCRL1 (EV—Figure 4 and Figure 5).

## 4. Conclusions

We previously indicated that mutations leading to perturbations in different parts of the OCRL1 molecule cause heterogeneity in terms of phenotype penetrance and the protein’s intracellular localization [30]. The current study points out that there are multiple levels of heterogeneity. even for mutations affecting a single domain of OCRL1.

Although more than 200 patient mutations have been identified all throughout the *OCRL1* gene, exons encoding for OCRL1′s phosphatase domain constitute a *hot spot* for disease-causative DNA alterations, and most correspond to missense mutations.

However, whether enzymatic function is more susceptible to being affected by, or more tolerant to, changes in specific regions or positions was unknown. Further, it is not clear what level of deviation from normal protein function, or degree of compromise in a biological process is tolerable vs. detrimental for cell and organismal physiology. Indeed, such missing information could help us to understand the complexity of the LS outcomes, and the observation that different mutations cause various degrees of symptom severity in different patients. 

In silico, we evaluated the pathogenic characteristics and the conformational characteristics of selected variants bearing residue changes in the phosphatase domain. Such studies revealed heterogeneity in the impact of different *OCRL1* mutations, with some being considered benign and likely to be normal population variations, while others were expected to be pathogenic. Specific structural alterations within the phosphatase domain were also identified. 

Next, we proceeded to evaluate the enzymatic activity and cellular function of the different OCRL1^VAR^. Specifically, we purified the different full-length OCRL1^VAR^ from human cells, measured their phosphatase activity in vitro and found that some were functional while others were unable to remove the 5′ phosphate group from their substrate PI(4,5)P_2_. It should be noted that this is the first study to systematically analyze the enzymatic activity of a battery of purified (from a LS-relevant kidney cells) OCRL1 variants in vitro, i.e., in the absence of confounding factors, such as other enzymes and multiple confounding factors present in lysates from fibroblasts.

Based on their enzymatic activity and induction of Golgi apparatus fragmentation, the OCRL1 variants were segregated in two categories:

Group 1: p.K293E, p.M299I, p.R318H, p.D431N and p.I533T: These variants were characterized by having normal Golgi apparatus morphology and measurable OCRL1 phosphatase activity. It should be noted that p.M299I and p.D431N are likely to be benign/normal population variations, while the others have been linked to the milder, LS-related condition known as Dent-2 disease.

Group 2: p.I393F, p.D451G, p.V508D and p.Y513C induced Golgi apparatus fragmented morphology and lacked OCRL1 phosphatase activity. Except for p.I393F, these variants have been linked to full blown LS. Indeed, our work also indicates that cells expressing these OCRL1^VAR^ yielded more severe phenotype (i.e., Golgi complex fragmentation) than cells lacking OCRL1 (i.e., *OCRL1* K.O. cells transfected with empty vector EV—see Figure 4 and Figure 5). Nevertheless, complementary studies using other (e.g., brain- and eye-derived) cell types and different genetic backgrounds (as well as more sophisticated models) will be necessary before robustly predicting organ/organismal impact for patients. Even for prognostics of kidney function, the impact of *OCRL1* mutations may require testing other renal-relevant processes, such as oxalate crystal clearance and proximal tubular function in general.

**Figure 6:**
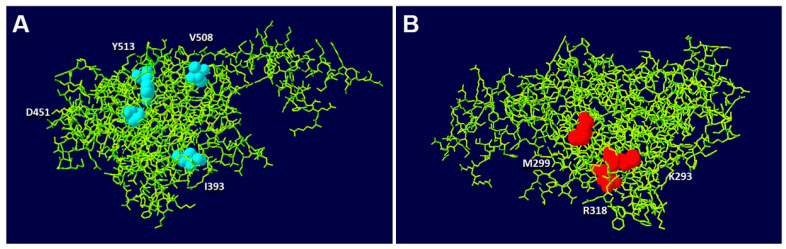
Residues affected by missense mutation spatially segregate according to biochemical impairment and phenotype severity. Residues affected by mutations from group 2 (**A**) and some of those in group 1 (**B**) (see text for details), were mapped on the 3D structure of the phosphatase domain of OCRL1 (PDB file 4CMN). Panels A and B correspond to two views of the OCRL1 phosphatase domain crystal structure after a rotation of ≈180 degrees.

Interestingly, substituted residues in the latter group cluster together on one side of OCRL1′s phosphatase domain, while several amino acids changed by missense mutations in group 1 are located on the opposite side of the structure (Figure 6). It should also be mentioned that alternative patient mutations affecting the same positions yielded identical effect on Golgi complex morphology (Supplementary Figure S7). These results suggest that certain regions within the domain are less tolerant to alterations. Expanding these studies to more variants (bearing different substitutions at the same and different positions) would lead to better definition of such change-sensitive/change-tolerant regions, or perhaps to identification of other similar residue clusters.

Indeed, this article describe the basis for a genotype to phenotype correlation: knowing in what overall region of the domain the mutation causes residue change allows predictions in terms of disease severity. 

A corollary of our study is that some disease causative mutations (even when triggering the milder D2 version of the disease) can display normal phosphatase activity and normal Golgi complex morphology (group 1); this observation implies that such variants must trigger other abnormalities and/or may greatly affect other cell types and/or be more sensitive to specific genetic backgrounds. This topic should be further studied to better understand details concerning the requirements for normal physiological function of OCRL1 and LS mechanistic details. Further, this knowledge is of great importance for the design and implementation of therapeutic strategies.

In summary, this manuscript focuses on the structural, biochemical and phenotypic consequences of some reported *OCRL1* mutations affecting the phosphatase domain but not directly altering its catalytic site. Our findings highlight that not every mutation located within the catalytic domain impairs the enzymatic activity of OCRL1. Importantly, alterations at positions outside the active site can affect OCRL1′s catalytic function and support the idea of mutation-stabilized non-functional conformations (i.e., the existence of a conformational disease component for LS). This study identified specific regions/positions less tolerant to missense mutations. Further, our work points out to the fact that certain mutations can be more deleterious than the absence of OCRL1.

Overall, our results further contribute to establishing the structural and molecular basis for the observed heterogeneity in severity and symptomatology displayed by patients.

## Figures and Tables

**Table 1:** Selected OCRL1 Variants Used in This Study.

Variant	p.H524R	p.K293E	p.M299I	p.R318H	p.I393F	p.D431N	p.D451G	p.V508D	p.Y513C	p.I533T
**Condition linked to ^a^**	LS	D2	Not Specified	LS/D2	D2	Not Specified	LS	LS	LS	D2
**Pathogenicity** **Prediction ^b^**	P	P	Benign	P	P	Benign	P	P	P	P
**Normalized Median Fluctuation ^c^**	0.99	0.93	1.10	1.30	0.77	1.33	1.26	2.22	0.97	0.83
**Missensense-3D ^d^**	NSD	NSD	NSD	NSD	NSD	NSD	SD	NSD	NSD	NSD

**a**: Lowe syndrome (LS), Dent-2 disease (D2). **b**: Pathogenic (P). **c**: The median fluctuation throughout all residues within the phosphatase domain was calculated using Molecular Dynamics (see text for details) and normalized with respect to the median fluctuation value exhibited by the WT domain (3.31 Å). **d**: Structural prediction. No structural damage (NSD), Structural damage (SD).

## Data Availability

The data presented in this study are available in the article and supplementary material.

## Supplementary Materials

The following supporting information can be downloaded at: https://www.mdpi.com/article/10.3390/biom13040615/s1, Figures S1–S5: Structural impact of each variant’s amino acid substitution; Figure S6: Stability and expression levels of some GFP-Ocrl1^WT/VAR^; Figure S7: Presence and magnitude of the Golgi apparatus fragmentation phenotype induced by variants displaying alternative substitutions; Table S1: OCRL1 Variant’s Complete Pathogenicity Results; Table S2: Antibodies; Table S3: Plasmids.
