# Supplementary material for: Heterogeneity in Lowe Syndrome: Mutations Affecting the Phosphatase Domain of OCRL1 Differ in Impact on Enzymatic Activity and Severity of Cellular Phenotypes
Source: Biomolecules. 2023 Mar 29;13(4):615. doi: 10.3390/biom13040615 (PMC10135975; doi:10.3390/biom13040615)
Supplement: Supplementary file 1 [file biomolecules-13-00615-s001.zip › Supplemental Material Lee et al Biomolecules 2023_revised.pdf]

SUPPLEMENTAL MATERIAL

Supplemental Table S1: OCRL1 Variant's Complete Pathogenicity Results. Is provided as an additional worksheet file with a tab containing analysis results ("OCRL1 Variants") and another with a brief explanation of the tool output ("Description of Utilized Tools").

Supplemental Table S2: Antibodies			
**Antigen**	**Host**	**Source**	**Working Dilution**
Acetylated-Tubulin	Mouse	Sigma Aldrich (6-11B-1)	1:1000[a]
Pericentrin-2 (PC-2)	Rabbit	Abcam (ab4448)	1:300[a]
TGN46	Rabbit	Thermo Fisher (PA5-23068)	1:400[a]
GFP	Rabbit	Thermo Fisher (A-11122)	1:1200[b]
His	Mouse	Thermo Fisher (MA1-21315)	1:1000[b]
Ocrl1	Mouse	Santa Cruz (sc-393577)	1:750[b]
Tubulin	Mouse	Biolegend (627903)	1:500[b]
a: Immunofluorescence.<br>b: Western Blot.			

Supplemental Table S3: Plasmids		
**Plasmid Name**	**Description**	**Source**
pEGFP-EV	pEGFP-c1 empty vector	Clontech
pEGFP-Ocrl1$^{WT(b)}$	GFP-Ocrl1$^{1\text{-}893 (isoform\ b)}$ in pEGFP-c1	Coon et al. 2009
pEGFP-Ocrl1$^{K293E(b)}$	GFP-Ocrl1$^{1\text{-}893,\ K293E}$ in pEGFP-c1	This study
pEGFP-Ocrl$^{M299I(b)}$	GFP-Ocrl1$^{1\text{-}893,\ M299I}$ in pEGFP-c1	This study
pEGFP-Ocrl1$^{R318H(b)}$	GFP-Ocrl1$^{1\text{-}893,\ R318H}$ in pEGFP-c1	This study
pEGFP-Ocrl1$^{D431N(b)}$	GFP-Ocrl1$^{1\text{-}893,\ D431N}$ in pEGFP-c1	This study
pEGFP-Ocrl1$^{D451G(b)}$	GFP-Ocrl1$^{1\text{-}893,\ D451G}$ in pEGFP-c1	Ramadesikan et al. 2021
pEGFP-Ocrl1$^{V508D(b)}$	GFP-Ocrl1$^{1\text{-}893,\ V508D}$ in pEGFP-c1	Ramadesikan et al. 2021
pEGFP-Ocrl$^{Y513C(b)}$	GFP-Ocrl1$^{1\text{-}893,\ Y513C}$ in pEGFP-c1	This study
pEGFP-Ocrl1$^{I533T(b)}$	GFP-Ocrl1$^{1\text{-}893,\ I533T}$ in pEGFP-c1	This study
pEGFP-Ocrl1$^{H524R(b)}$	GFP-Ocrl1$^{1\text{-}893,\ H524R}$ in pEGFP-c1	Ramadesikan et al. 2021
pNIC-CH2-Ocrl1$^{WT(5P)}$	Ocrl1$^{215\text{-}563}$-His in pNIC-CH2	This study (modified from Trésaugues et al. 2014)
pNIC-CH2-Ocrl1$^{WT(5P)}$	Ocrl1$^{215\text{-}563}$-His in pNIC-CH2	This study
pNIC-CH2-Ocrl$^{M299I(5P)}$	Ocrl1$^{215\text{-}563,\ M299I}$-His in pNIC-CH2	This study
pNIC-CH2-Ocrl1$^{R318H(5P)}$	Ocrl1$^{215\text{-}563,\ R318H}$-His in pNIC-CH2	This study
pNIC-CH2-Ocrl1$^{D431N(5P)}$	Ocrl1$^{215\text{-}563,\ D431N}$-His in pNIC-CH2	This study
pNIC-CH2-Ocrl1$^{D451G(5P)}$	Ocrl1$^{215\text{-}563,\ D451G}$-His in pNIC-CH2	This study
pNIC-CH2-Ocrl1$^{V508D(5P)}$	Ocrl1$^{215\text{-}563,\ V508D}$-His in pNIC-CH2	This study
pNIC-CH2-Ocrl$^{Y513C(5P)}$	Ocrl1$^{215\text{-}563,\ Y513C}$-His in pNIC-CH2	This study
pNIC-CH2-Ocrl1$^{I533T(5P)}$	Ocrl1$^{215\text{-}563,\ I533T}$-His in pNIC-CH2	This study
pNIC-CH2-Ocrl1$^{H524R(5P)}$	Ocrl1$^{215\text{-}563,\ H524R}$-His in pNIC-CH2	This study

Supplemental Figure S1

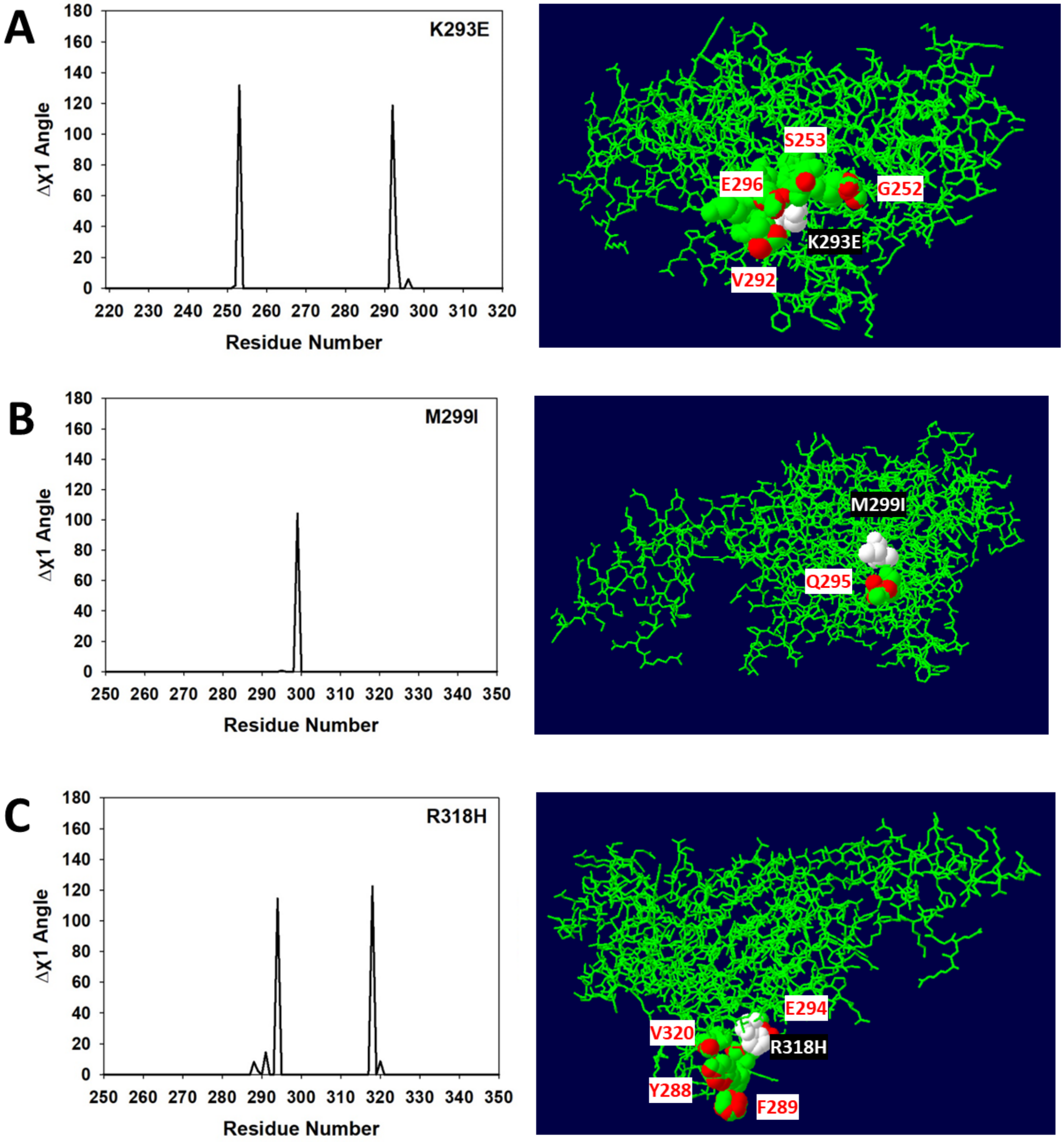

Supplemental Figure S1 (Cont'd)

D

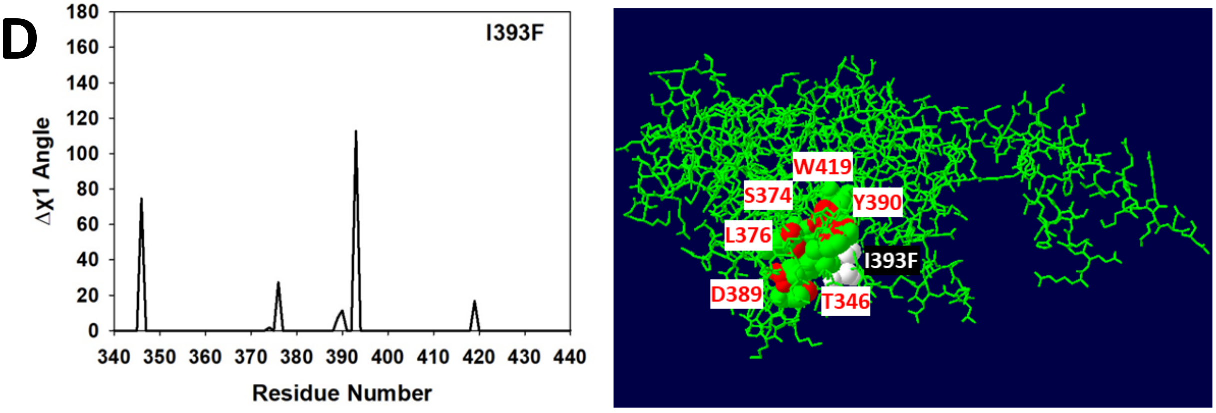

E

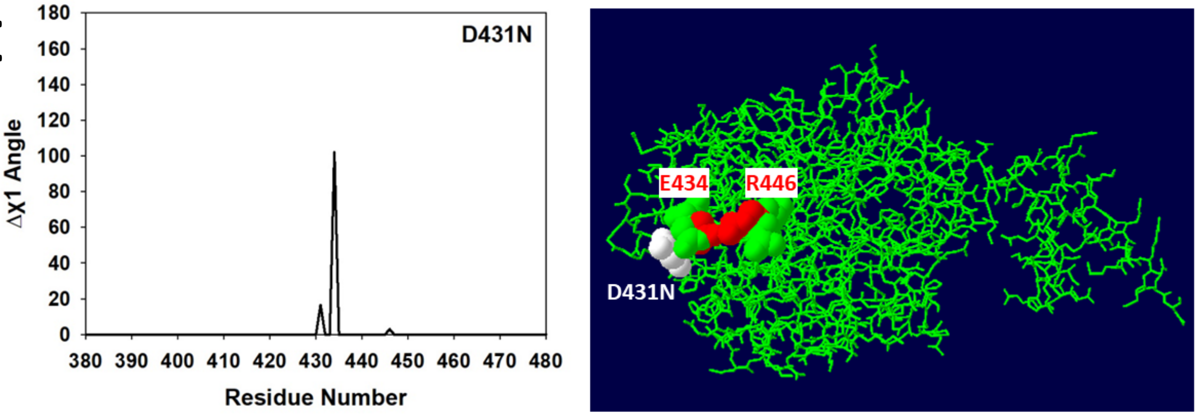

In addition to changes in residue mobility (Fig.2, main text), This figure provides examples of the changes in residue spatial position due to mutation-encoded amino acid substitution for different variants (**A-D**). *Left panels*: as a way of quantifying such changes, we calculated the N-Cα-Cβ-Cγ torsion angles $\chi1$ (Thomas E. Creighton (1983). *Proteins: structures and molecular principles*. New York: W.H. Freeman) for every residue within the variants' phosphatase and WT's domains. Y-axis represents the *absolute* value of the difference between torsion angles of residues within each variant's and the WT's catalytic domain; X-axis shows the range of residues in which a change of the $\chi1$ angle was detected. *This also includes differences in side-chain's space use by the variant's substituted residue*. Python code used for such calculations is available as part of the supplemental materials. *Right panels*: The 3D structure of the WT OCRL1 phosphatase domain (green-4CMN) is shown overlapped on top the variant's (red-produced by Missense3D). Therefore, red variant's residues can only be visible if they have a different spatial orientation with respect to the corresponding green WT's amino acids. In those cases, both variant's and WT's residues were presented in space-filled mode and a red label was added. Residue substitution is also shown in white color and space filled mode accompanied by a white label. Representations were made using Swiss PDB Viewer. Note that in panel **D** the p.I393F variant shows a change in the spatial orientation of S374 which is a central residue in one of the important catalytic boxes of OCRL1's catalytic site (See Fig.1, main text) consistent with its deficient phosphatase activity.

Supplemental Figure S2

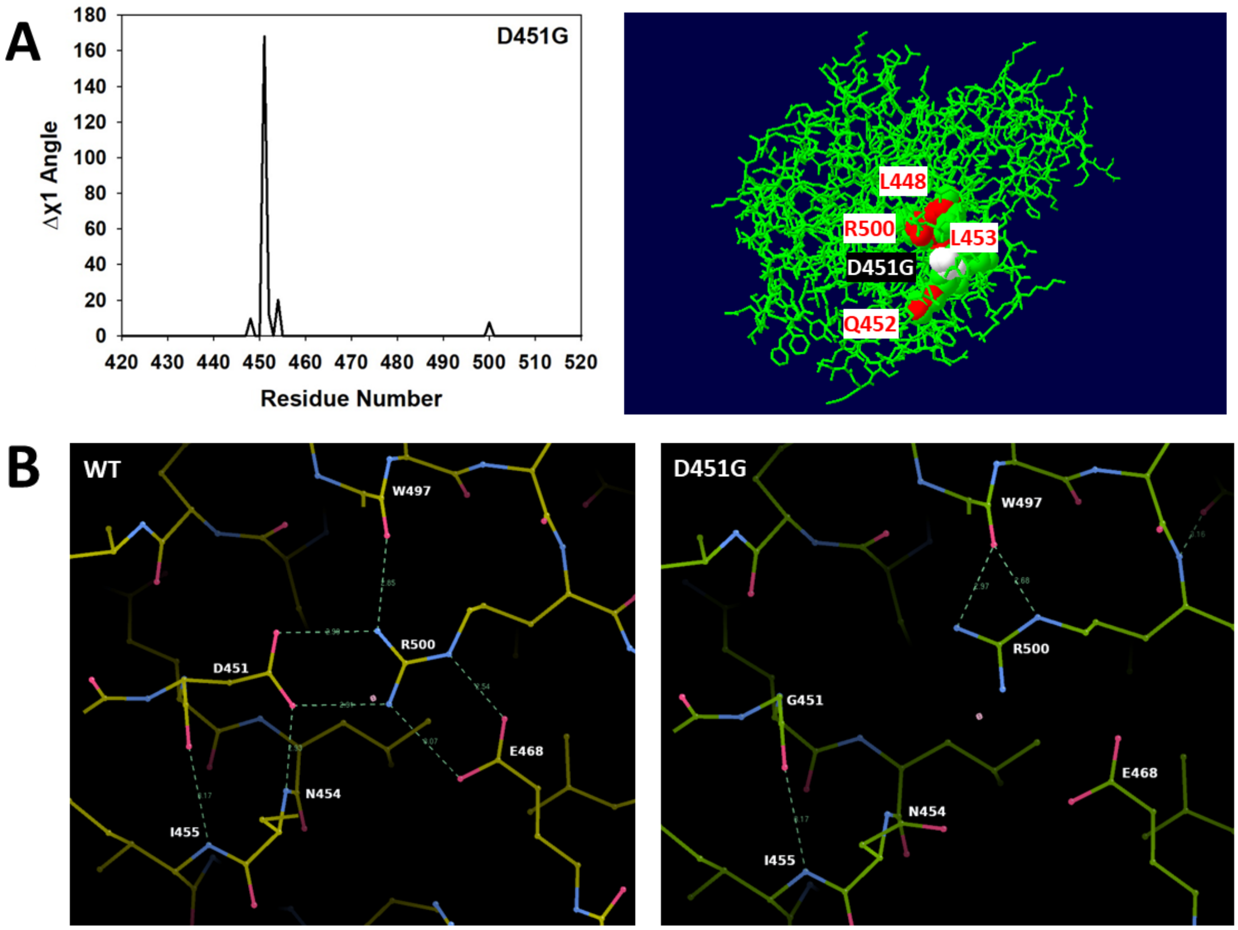

A. See legend to Supplemental Figure S1. **B.** Enlargement of the region surrounding D451 in WT OCRL1 (left) and the variant displaying the D451G substitution (right) are shown from Missense3D-generated structure using *Coot* (Emsley, P., & Cowtan, K. (2004). Coot: model-building tools for molecular graphics. *Acta crystallographica section D: biological crystallography*, *60*(12), 2126-2132). D451 caps an alpha helix and is involved in a tight hydrogen bonding network (left). This whole network is significantly disrupted by substitution to glycine, as seen in the right panel. R500 adopts an entirely different rotamer. These changes cause an overall loss of stability by removing a key interaction between a helix (D451) and a beta strand (R500).

Supplemental Figure S3

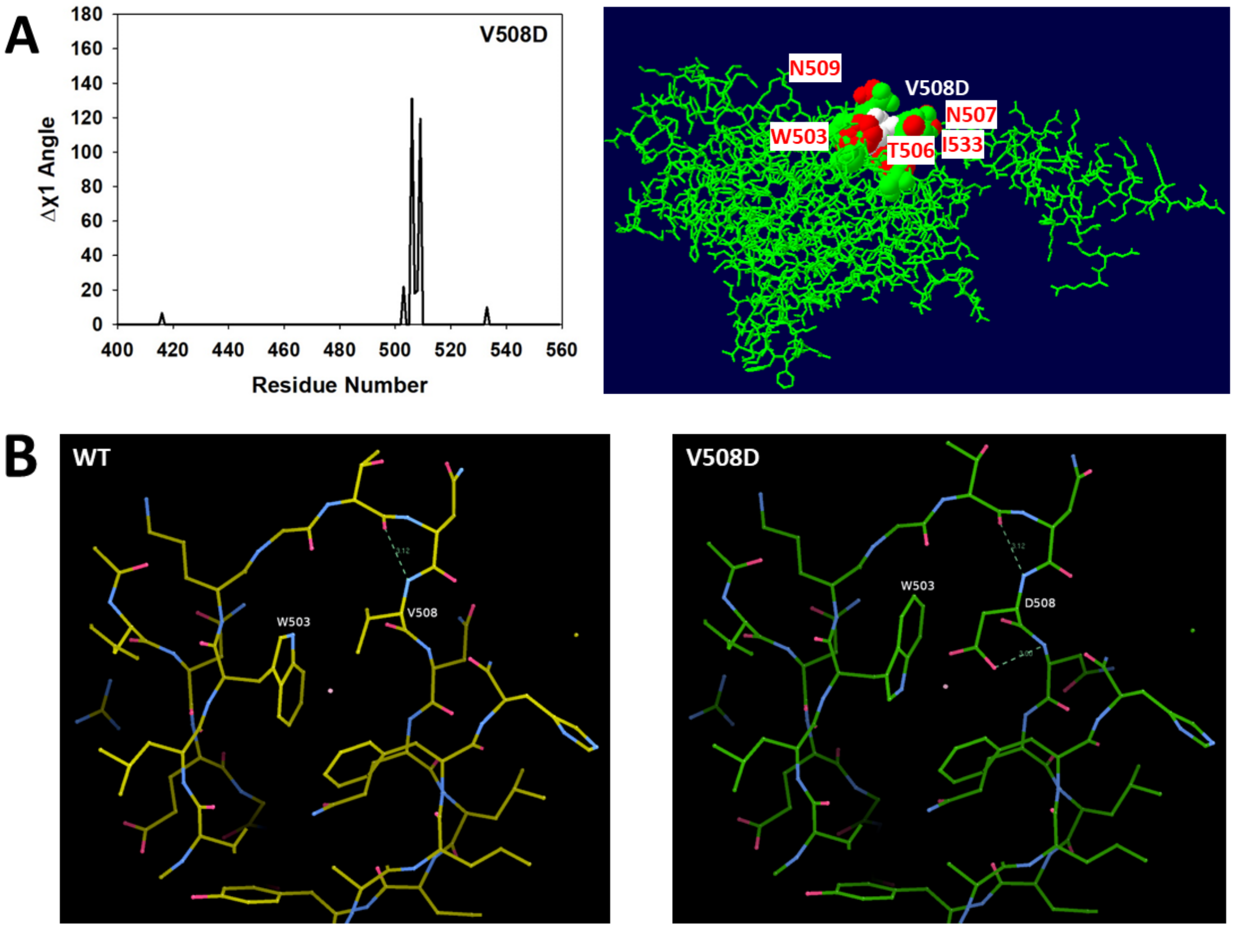

A. See legend to Supplemental Figure S1. **B.** Enlargement of the region surrounding V508 in WT OCRL1 (left) and the variant displaying the V508D substitution (right) are shown from Missense3D-generated structure using Coot. It should be noted that V508 is located on a loop that connects two of the beta strands adjoining the substrate binding and catalytic regions. Disruption of this region significantly impact subtle backbone conformations required for productive active site organization. *Left*: In $OCRL1^{WT}$ there is one backbone stabilizing hydrogen bond (3.12 Å). *Right*: the V508D permutation places a bulkier, polar side chain into a tight and relatively hydrophobic space. The rotamer forms an additional hydrogen bond (3 Å) with the backbone and generates a near clash with the side chain of W503, forcing a flip that leaves a distance of only ~3Å between the two. There is no conformation that relieves steric hindrance without backbone movement.

Supplemental Figure S4

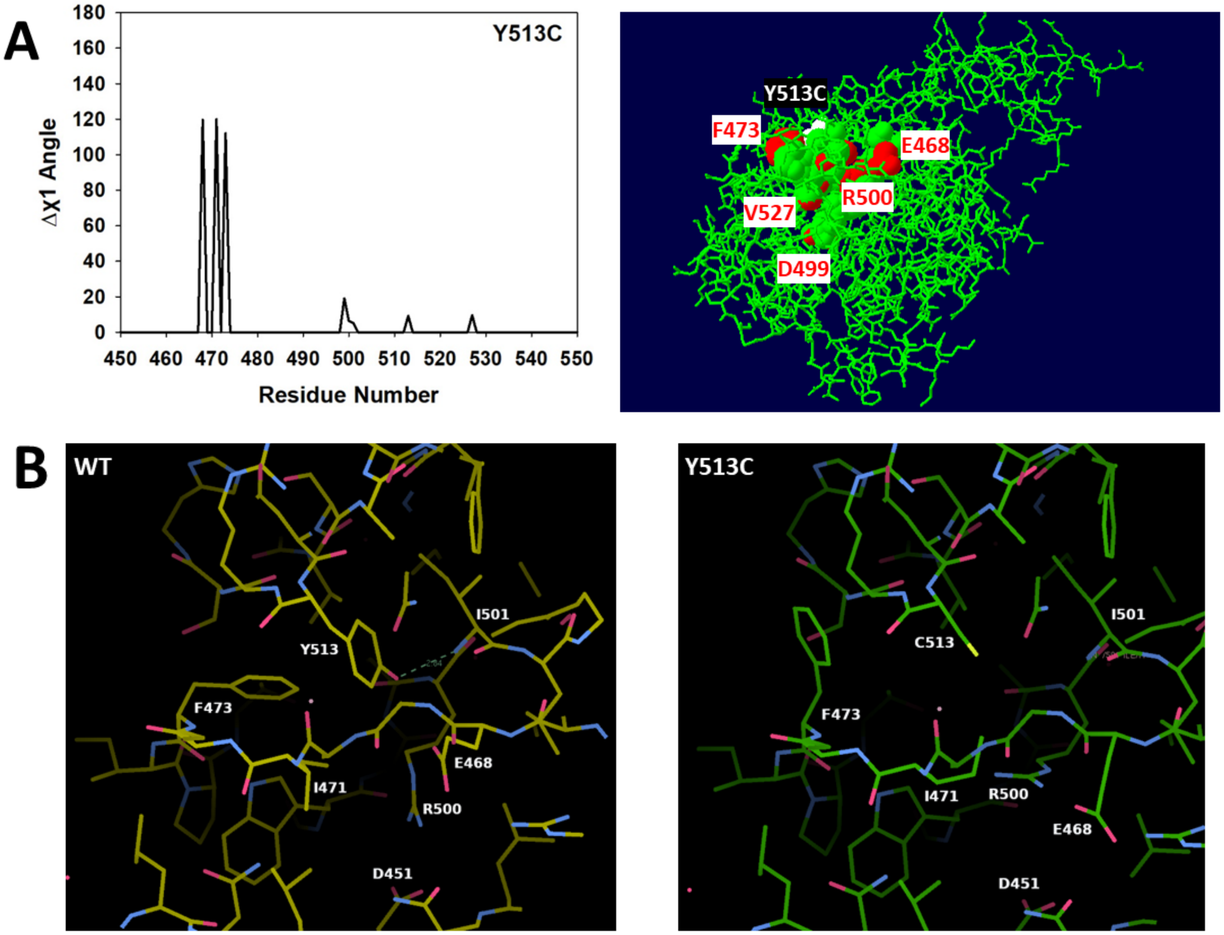

A. See legend to Supplemental Figure S1. **B.** Enlargement of the region surrounding Y513 in WT OCRL1 (left) and the variant displaying the Y513C substitution (right) are shown from Missense3D-generated structure using Coot. The Y513C substitution leads to several significant rotamer changes in surrounding residues (F473, R500, E468) and breaks a hydrogen bond between Y513 and the backbone of I501. This creates a hole in this region (left vs right panel). Notably, R500 gains the freedom to adopt alternative rotamers (coincident with predictions of abnormally high residue mobility, Fig.2 main text). The prediction of the mutant structure in Missense3D indicates this flexibility in R500 is also likely to disrupt the salt bridge with D451 mentioned in Supplemental Fig.S2.

Supplemental Figure S5

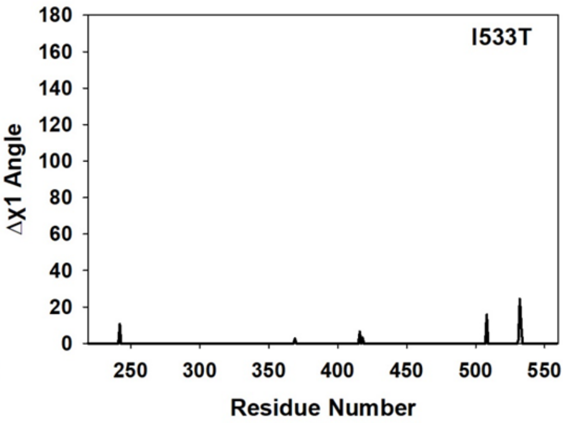

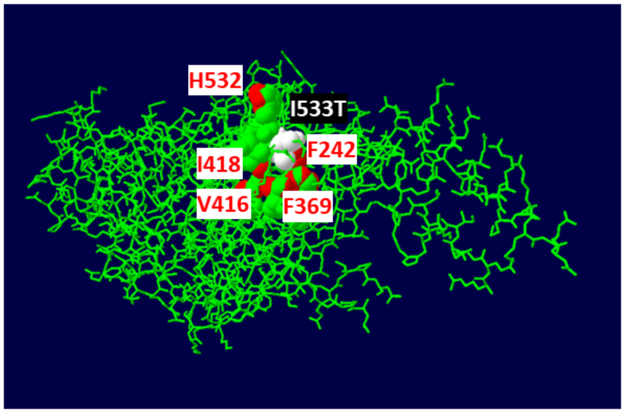

See Legend to Supplemental Fig. S1.

Supplemental Figure S6:

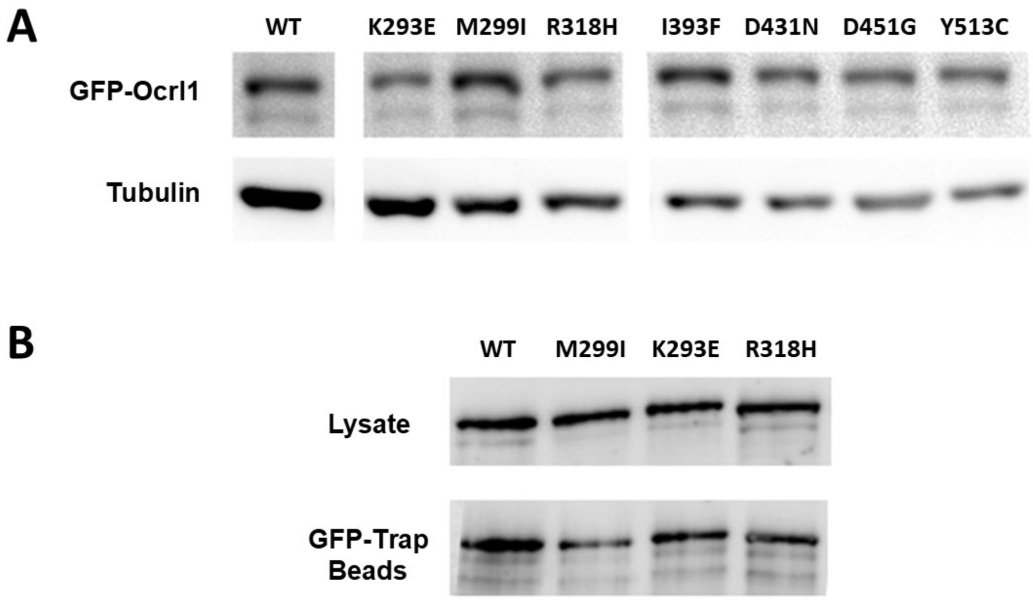

The stability and expression levels of some GFP-Ocrl1$^{WT/VAR}$ (**A**) as well as the loading of GFP-Trap beads with the variants under study (only 4 are shown as example: **B**) were monitored by Western blotting. WB was conducted as described in *Materials and Methods* on whole cell lysates. The presence of GFP-Ocrl1$^{WT/VAR}$ was investigated using an anti-GFP antibody (**A** and **B**), while the signal from tubulin was used as loading control (**A**).

Supplemental Figure S7

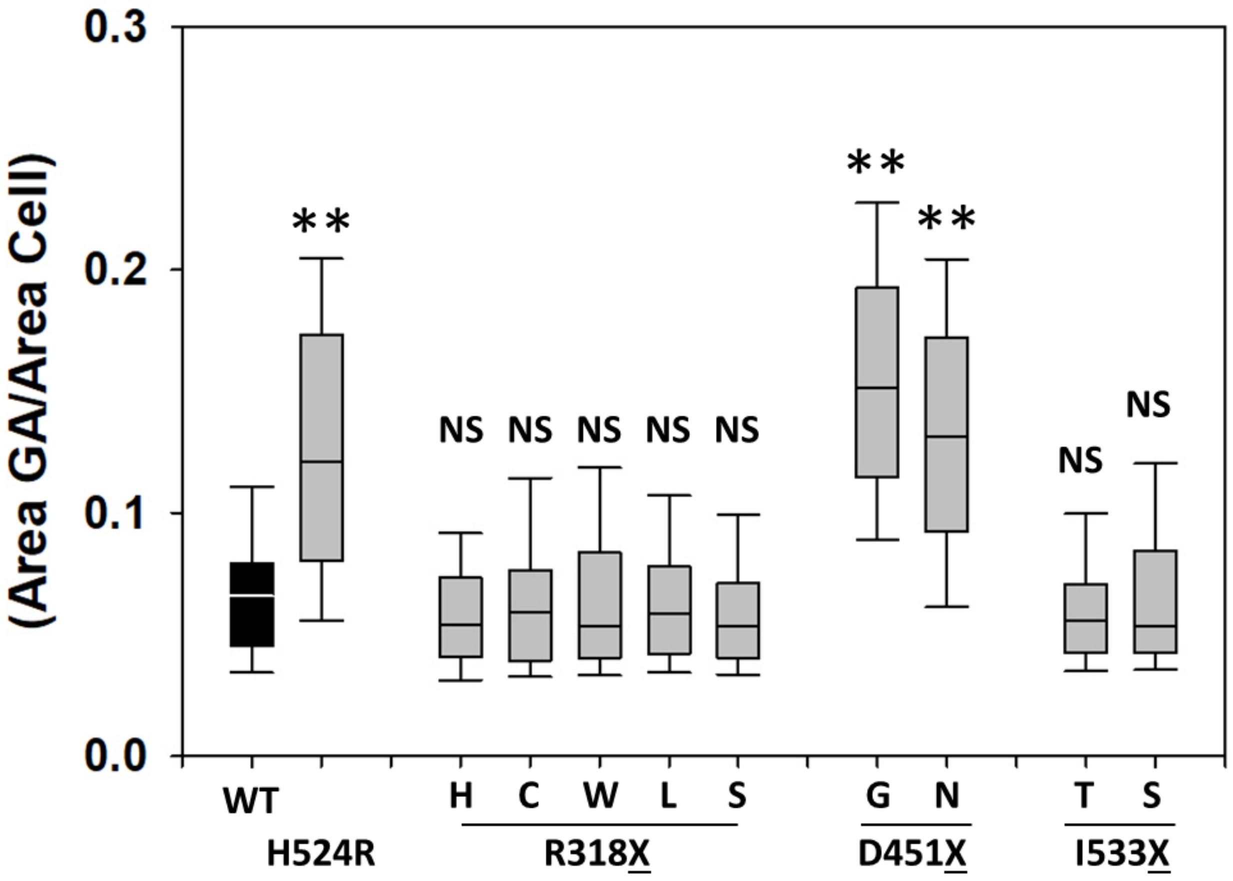

Presence and magnitude of the Golgi apparatus fragmentation phenotype induced by variants displaying alternative substitutions at the same position was assessed by computing the ratio between the area occupied by the Golgi apparatus (GA) and the one corresponding to the whole cell (see main text for details). Different substitutions are indicated by a specific residue sequence position (*e.g.,* R318; D451; I533) followed by a variable amino acid (X) resulting from different missense *OCRL1* mutations observed in patients at such position. Different X tested are shown above the specific residue (*e.g.,* R318X was tested as the R318H, R318C, R318W, R318L and R318S patients' substitutions—see Fig.1). WT and H524R were used as negative and positive controls, respectively. Statistical significance of the differences between the distributions of WT and variants was assessed using the Wilcoxon test with $p<0.05$ applying Bonferroni's correction for 10 comparisons $\alpha_B \leq 0.005$ (**) per comparison. NS: No significant.
